# A mulberry 9-*cis*-epoxycarotenoid dioxygenase gene *MaNCED1* is involved in plant growth regulation and confers salt and drought tolerance in transgenic tobacco

**DOI:** 10.3389/fpls.2023.1228902

**Published:** 2023-07-28

**Authors:** Panpan Zhu, Ruolan Li, Wei Fan, Zhongqiang Xia, Jun Li, Chuanhong Wang, Aichun Zhao

**Affiliations:** ^1^State Key Laboratory of Resource Insects, Institute of Sericulture and Systems Biology, Southwest University, Chongqing, China; ^2^Resource Institute for Chinese & Ethnic Materia Medica, Guizhou University of Traditional Chinese Medicine, Guiyang, China; ^3^The National Engineering Laboratory of Crop Resistance Breeding, School of Life Sciences, Anhui Agricultural University, Hefei, China

**Keywords:** mulberry NCED, plant hormones, abiotic stress, seed germination, root elongation

## Abstract

The phytohormone abscisic acid (ABA) is vital in regulating root elongation, seed germination, and abiotic stress responses in plants. Conversely, the mechanisms of ABA in mulberry root growth, seed germination, and abiotic stress responses are poorly understood. Here, we reported that exogenous ABA and drought treatment inhibited the growth of mulberry seedlings but significantly increased the ratio of root/stem. Inhibition of ABA synthesis by fluridone and sodium tungstate resulted in the decrease of root/stem ratio. We also showed that the expression of *MaNCED1* in the root was strongly induced by drought and salt stress. Increasing the expression of *MaNCED1* in tobacco using overexpression leads to increased root elongation and reduced seed germination. Compared with the wild type, the accumulation of H_2_O_2_ and MDA was reduced, while the POD activity and proline content was increased in the transgenic plants after drought and salt treatment. Further studies revealed increased resistance to drought and salt stress in *MaNCED1* overexpressed tobaccos. Meanwhile, the auxin and ethylene signal pathway-related gene expression levels increased in *MaNCED1* overexpressed tobaccos. This study demonstrated the roles of mulberry *MaNCED1* in regulating plant development and abiotic stress responses. It gave further insights into the coordinated regulation of ABA, auxin, and ethylene in seed growth and germination.

## 1 Introduction

Abiotic stress was one of the most critical factors to affect plant development and growth. Every year many crops growing worldwide suffers disasters caused by drought and soil salinity (Golldack et al., 2014; Guo et al., 2014; Zandalinas et al., 2018; Molnár et al., 2021). Currently, the lands under drought and salinity stresses increased continuously. Abiotic stress affects plants’ physiological and biochemical processes (Araújo et al., 2011; Gonzalez-Villagra et al., 2022). Consequently, crop yields were reduced dramatically (Perez-Alfocea et al., 1993; Venkatappa et al., 2021). The plant had developed an effective mechanism to survive in an adverse environment with long-term evolution (Zhu, 2002; Krasensky and Jonak, 2012). Abscisic acid (ABA), as a plant hormone, plays an essential role in regulating plant growth, development, seed germination (Son et al., 2016), fruit ripening (Gan et al., 2020), and stress resistance (Liu et al., 2021; De Oliveira et al., 2022). In *stylosanthes guianensis*, the enzyme activity of SOD and APX were increased after ABA treatment (Zhou and Guo, 2005). However, the improvement activity of SOD and APX were suppressed after being treated with ABA biosynthesis inhibitor-sodium tungstate. Meanwhile, the contents of endogenous ABA were decreased (Zhou and Guo, 2005). In *Zea mays* and *Oryza sativa*, ABA treatment could improve the resistance to cold stress (Anderson et al., 1994; Huang et al., 2021). In addition, studies have found that ABA content is associated with seed dormancy (Wang et al., 2020).

ABA can not only inhibit plant growth but also can restrain seed dormancy. Previous research has shown that mulberry seedlings treated with exogenous ABA can shorten seedling root lengths, and the inhibition was increased along with the ABA concentration (Liu et al., 2014). In *Solanum lycopersicum*, exogenous ABA treatment inhibits plant growth and reduces lateral root growth, improving the root-to-shoot ratio (Hooker and Thorpe, 1998). The expression levels of some lateral root initiation genes were reduced in seedlings after being treated with the ABA. These genes were increased after auxin induction, indicating that ABA and auxin play antagonism roles in lateral root development (Zhang et al., 2009).

The biosynthesis pathway of ABA is well understood in higher plants. Two possible routes for ABA biosynthesis have been suggested, including direct and indirect ones (Seo and Koshiba, 2002). Studies have revealed that the biosynthesis of ABA in higher plants primarily *via* an indirect pathway, the synthesis of ABA begins with the production of violaxanthin catalyzed by zeaxanthin epoxidase from C40 carotenoids. Violaxanthin was cleavaged by 9-*cis*-epoxycarotenoid dioxygenase (NCED) to generate 9-*cis*-neoxanthin. Finally, 9-*cis*-neoxanthin was catalyzed by aldehyde oxidase and formed ABA (Seo and Koshiba, 2002; Xiong and Zhu, 2003). NCED is the critical enzyme in ABA synthesis. Treating with an NCED specificity inhibitor, nordihydroguaiaretic acid (NDGA) decreased endogenous ABA contents (Zhang et al., 2009).

Since the first *NCED* gene was isolated from the maize *vp14* mutant (Tan et al., 1997), it has been cloned in various plant species, such as *avocado*, *Arabidopsis thaliana*, and *Solanum lycopersicum* (Burbidge et al., 1999; Chernys and Zeevaart, 2000; Iuchi et al., 2001; Estrada-Melo et al., 2015). In abiotic stress conditions such as salt, drought, and heat, the same up-regulate pattern was found in *NCEDs* (Xia et al., 2014; Hwang et al., 2018). In cucumber, the expression of *CsNCED1* and *CsNCED2* was up-regulated on the third day after treatment with exogenous ABA, and the expression of *CsNCED1* and *CsNCED2* was up-regulated after water stress treatment (Wang et al., 2013). A similar increased expression was found in sweet cherry and Malus domestica after drought treatment (Ren et al., 2010; Kondo et al., 2012). In *A. thaliana*, drought stress induced the expression of the *AtNCED3* gene, which plays a vital role in ABA biosynthesis. Overexpression of the *AtNCED3* gene increased ABA content in *A. thaliana*, promoted the expression of drought- and ABA-induced genes, reduced the transpiration rate, and increased resistance to drought (Iuchi et al., 2001). The plants showed high sensitivity to drought in *AtNCED3*-silenced transgenic *A. thaliana* (Iuchi et al., 2001).

Mulberry is an important economic tree in China. The leaves are the main feed of silkworms, and the fruit has high edible and medicinal value for its abundant nutrients, active substances, and good mouthfeel. Additionally, mulberry was also used for ecological control due to its strong ability to resist stresses. ABA is central to regulating plant development and stress tolerance (Xiong and Zhu, 2003). However, the function of *MaNCED1* in mulberry development and stress response processes has not been reported to date. In this study, we studied the effects of different treatments on the growth of mulberry seedlings and analyzed the expression of the *MaNCED1* gene under various stresses. In addition, the drought and salt stress resistance ability was evaluated in the ectopic expression of *MaNCED1* transgenic tobacco.

## 2 Materials and methods

### 2.1 Plant material and treatments

Mulberry (*Morus atropurpurea* Roxb.) variety (Guisangyou 62) was selected as the experimental material. Seeds were soaked in sterile water at 4°C for 24 h, sown in a sterile petri dish lined with moist filter paper, and cultured at 25°C/22°C and 16 h light/8 h dark photoperiod incubator. After 7 days, the seedlings of Guisangyou 62 with uniform growth states were selected and treated with 60 μM fluridone, 100 mg/L ABA, 200 mM mannitol, 1 mM sodium tungstate, and 100 mM NaCl, respectively (Hooker and Thorpe, 1998; Zhang et al., 2009). Materials were collected 5 days after treatment.

To analyze the expression of *MaNCED1* under salt and drought conditions, the seedlings germinating for 7 days were selected and treated with 20% (w/v) PEG 6000 and 100 mM NaCl. Then, the seedlings were collected at 0 h, 1 h, 6 h, 12 h, 24 h, and 48 h after treatment. The roots and shoots were immediately separated with a scalpel, frozen in liquid nitrogen, and stored at -80°C for further use.

### 2.2 Determination of root and stem length of mulberry seedlings after treatment

To observe the effects of ABA, fluridone, sodium tungstate, NaCl, and mannitol treatments on mulberry seedling growth, the root and stem lengths of the seedlings treated for 5 days were measured by a ruler.

### 2.3 Cloning and bioinformatics analysis of *MaNCED1* gene

According to the manufacturer’s procedures (Invitrogen, Carlsbad, CA, USA), total RNA was extracted from mulberry seedlings and tobacco. The total RNA was used as a template to synthesize the first strand of cDNA using PrimeScript™ RT Reagent Kit (Takara Bio., Shiga, Japan). The primers were designed according to the sequence obtained from the *M. notabilis* genome database ((http://morus.swu.edu.cn/morusdb/). The complete *MaNCED1* gene coding sequence was obtained from Guisangyou 62 cDNA. Using the deduced amino acid sequences of MaNCED1 as queries to search in National Center for Biotechnology Information and obtained homolog amino acid sequences from other plant species (http://blast.ncbi.nlm.nih.gov/Blast.cgi). Multiple sequence alignment was performed using ClustalX software. A MEGA 4.0 software was used to construct the phylogenetic tree with a neighbour-joining method.

### 2.4 Plasmid construction and plant transformation

The full-length coding sequence of *MaNCED1* from mulberry was cloned into the pLGNL expression vector by *KpnI* and *SpeI* restriction enzyme. Then, the recombinant plasmid was transformed into *Agrobacterium tumefaciens* strain LBA4404. The positive *A. tumefaciens* harbouring the *MaNCED1* plasmid was transformed into tobacco (K326) plants using a leaf disk co-cultivation method (Li, 2011). Positive transgenic tobacco was confirmed by target gene and kanamycin resistance gene PCR assay and β-D-glucosidase (GUS) staining. Additionally, the expression levels of *MaNCED1* in transgenic tobacco plants were detected by real-time quantitative reverse transcription PCR (qRT-PCR) analysis. The endogenous ABA content was determined as described by Zhu et al. (2017).

### 2.5 Determination of germination rate and growth analysis of transgenic tobacco

The collected WT and T2 generation transgenic seeds were germinated on a petri dish covered with absorbent cotton and filter paper. The germination rates were statistics at 3 days, 4 days, and 5 days, respectively. To observe the effects of overexpressed *MaNCED1* on tobacco growth, the seedlings were incubated in a petri dish for 10 days, and the root length, stem length, and ratio of root/stem were counted.

### 2.6 Stress treatment of transgenic tobacco

The five-week-old WT and transgenic seedlings were chosen and independently irrigated with 30% PEG and 200 mM NaCl for 14 d. Each treatment was replicated three times. After the stress treatment, 1 g of treated tobacco leaves were collected. According to the manufacturer’s instructions, the malonaldehyde (MDA), H_2_O_2_, proline contents, and peroxidase (POD) activity were measured using test kits (Jiancheng Bioengineering Institute, Nanjing, China). Additionally, the expression levels of *NtSOD* and *NtCAT* in PEG and NaCl-treated plants were analyzed by qRT-PCR.

### 2.7 Quantitative real-time PCR analysis

The specific primers were designed using NCBI Primer-BLAST (https://www.ncbi.nlm.nih.gov/tools/primer-blast/index.cgi) (**Supplementary Table 1**). The treated mulberry and tobacco cDNA served as the qRT-PCR template. qRT-PCR analysis was performed according to the instructions of the SYBR Green Reagent Kit (Takara Bio.). The test was performed on a StepOnePlus real-time PCR machine (ABI Company). *MaACTIN3* (HQ163776) and *NtActin* (U60489) were used as the internal control for mulberry and tobacco. The relative expression was calculated using the 2^-ΔΔCt^ method.

### 2.8 Statistical analyses

All the experiments in this study were repeated at least three times. The results were collected and calculated by Excel 2013 (Microsoft, Redmond, WA, USA). The final results are shown as means ± standard deviations (SD). Statistical analysis was performed by SPSS Statistics 17.0 software (SPSS Inc., Chicago, IL, USA). The graphs were created using GraphPad Prism 5 software (GraphPad Software Inc., La Jolla, CA, USA).

## 3 Results

### 3.1 Effects of ABA, fluridone, sodium tungstate, mannitol, and NaCl on the growth of mulberry seedlings

**Figure 1:**
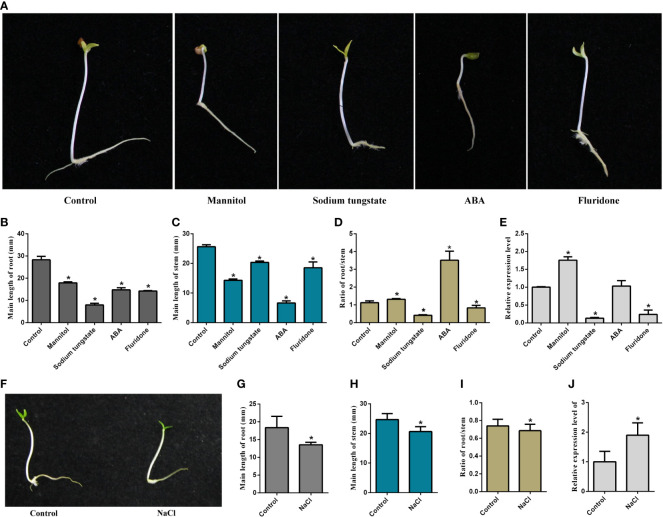
Effects of various reagents on mulberry seedlings growth. **(A, F)** Phenotypes change of seeding after being treated with mannitol, sodium tungstate, ABA, fluridone, ddH_2_O, and NaCl for 5 days; **(B, G)** Root length of seedlings after treatments; **(C, H)** Stem length of seedlings after treatments; (D, I) Ratio of root/stem of seedlings after treatments; **(E, J)** Expression analysis of *MaNCED1* gene in root after treatments. The data were indicated as mean ± SD from three replications (n=30). *p < 0.05.

The growth of the seedlings treated with ABA, fluridone, sodium tungstate, mannitol, and NaCl was inhibited in varying degrees, mainly manifested in the reduction of the average length of roots and stems compared with the control (**Figure 1**). However, the ratio of root/stem was significantly increased under mannitol and exogenous ABA treatments (**Figure 1D**). In contrast, the ratio of root/stem was decreased considerably after sodium tungstate, fluridone, and NaCl treatment (**Figures 1D, I**). We further examined the changes in gene expression of *MaNCED1* under different treatments. As shown in **Figures 1E, J**, the expression of *MaNCED1* increased significantly after mannitol and NaCl treatment and was only slightly induced after ABA treatment. Notably, treated with the ABA synthesis inhibitors, fluridone and sodium tungstate significantly reduced *MaNCED1* expression (**Figure 1J**).

**Figure 2:**
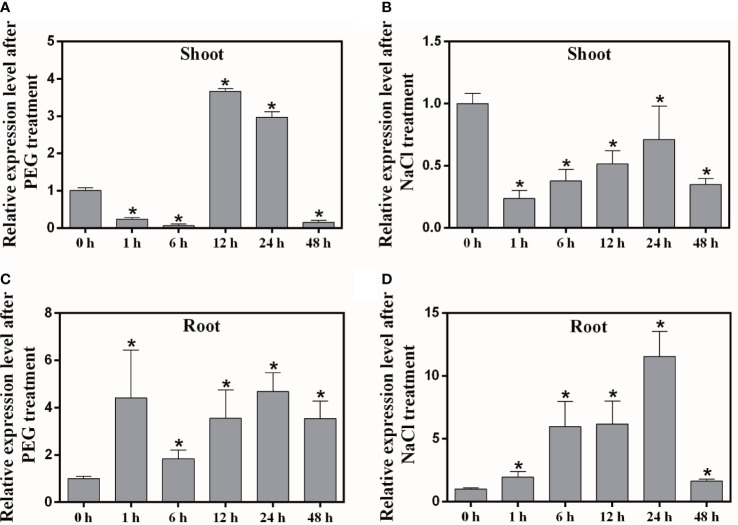
Expression analysis of *MaNCED1* gene in mulberry shoots and roots after PEG and NaCl treatments. **(A, B)** Transcription levels of *MaNCED1* gene in shoot after PEG and NaCl treatment for 0 h, 1 h, 6 h, 12 h, 24 h, and 48 h, respectively. **(C, D)** Transcription levels of *MaNCED1* gene in root after PEG and NaCl treatment for 0 h, 1 h, 6 h, 12 h, 24 h, and 48 h, respectively. The data were indicated as mean ± SD from three replications. Significant differences are marked with an asterisk (*p* < 0.05).

In order to understand whether the *MaNCED1* gene is involved in abiotic stress response, we detected the expression of *MaNCED1* after PEG and NaCl treatment. The expression level of *MaNCED1* decreased at 1 h in shoot after PEG and NaCl treatment and then up-regulation at 12 h and 24 h (**Figures 2A, B**). In the root, the transcript of *MaNCED1* was up-regulated by PEG and NaCl treatment and peaked at 24 h (**Figures 2C, D**). These results indicated that *MaNCED1* responded to drought and salt stress, and its expression was strongly induced in the root.

### 3.2 Phylogenetic and sequence alignment analysis of MaNCED1

The full-length sequence of the *MaNCED1* (GenBank accession number: KX181538.1) gene was obtained by amplification in the cDNA of Guisangyou 62. To examine the evolutionary relationship between MaNCED1 and other plant NCED proteins, we constructed a phylogenetic tree using MEGA 4.0 software. The results showed that these plant NCED proteins were classified into two main groups. The MaNCED1 is closely related to the NCEDs from *Malus domestican*, *Rosa chinensis*, and *Solanum lycopersicum* and belongs to dicotyledonous plants. The monocotyledonous plants such as *Z. mays*, *O. sativa*, *Sorghum bicolor*, *Setaria italic*, and *Triticum aestirum* were classified into the second group (**Supplementary Figure 1**). We further compared the amino acid sequences of MaNCED1 with other NCEDs. The amino acid sequence of MaNCED1 shared 76.92% identity with Citrus sinensis, 71.09% identity with Solanum lycopersicum, and 73.95% identity with Malus domestica. These four putative NCED proteins showed high similarity to each other and the same conserved structural domain (**Supplementary Figure 2**).

### 3.3 Determination of transgenic tobacco plants

**Figure 3:**
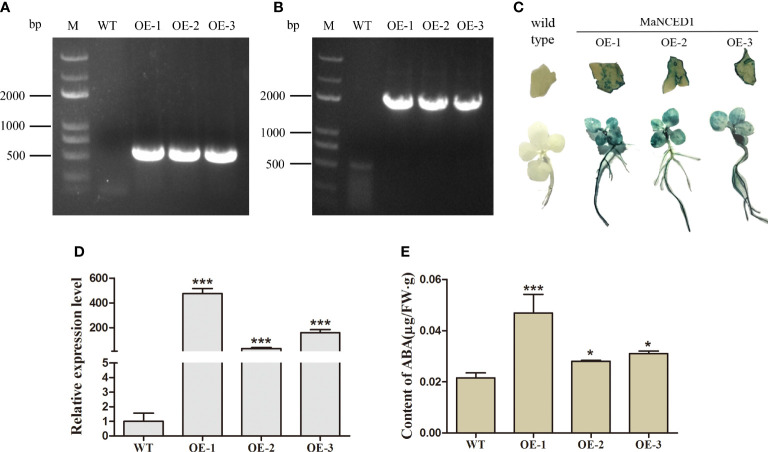
Confirmation of transgenic tobacco plants. **(A)** Amplification of the kanamycin resistance gene and **(B)** *MaNCED1* from the genome of transgenic plants. **(C)** Histochemical GUS staining of transgenic lines. **(D)** Quantitative real-time PCR analysis of *MaNCED1*. **(E)** ABA contents in WT and *MaNCED1* overexpressing tobaccos. Data represent the means ± SD (n = 3), *p < 0.05, ***p < 0.001.

To evaluate the roles of the *MaNCED1* gene in plant tolerance to drought and salt stress, the full-length sequence was cloned into the pLGNL expression vector and transformed into wild-type (WT) tobacco plants. Eventually, three independent transgenic plants were obtained using GUS staining and qRT-PCR analyses (**Figures 3A-D**). The expression level of *MaNCED1* increased significantly in transgenic plants and was 475.5 folds, 31.9 folds, and 158.3 folds higher than that of WT, respectively (**Figure 3D**). Meanwhile, the content of ABA in *MaNCED1* overexpressing plants was significantly higher than in WT, indicating that *MaNCED1* was overexpressed in tobacco successfully (**Figure 3C**).

### 3.4 Overexpression of *MaNCED1* reduces tobacco germination rate and promotes root growth

**Figure 4:**
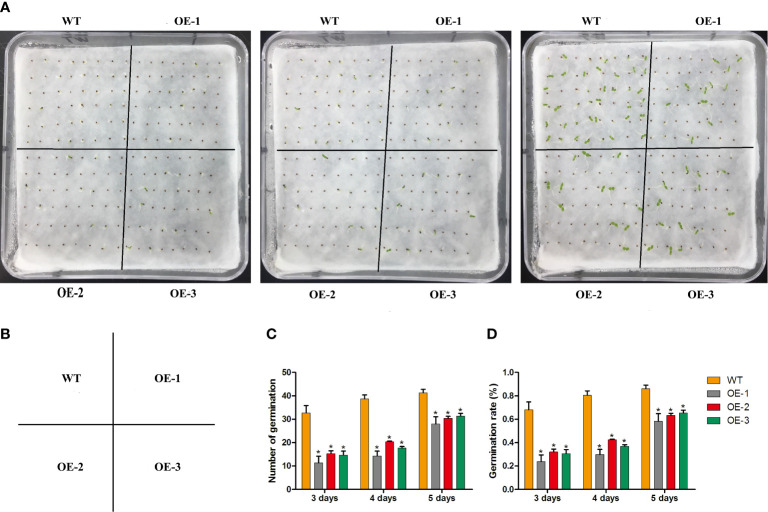
Statistical analysis of seed germination rates of WT and *MaNCED1* overexpression plants. **(A)** The germination phenotype of WT and *MaNCED1* overexpression plants. **(B)** The distribution diagram of WT and *MaNCED1* overexpression plants. **(C)** The number of germinated seeds and **(D)** seed germination rates of WT and *MaNCED1* overexpression plants. Seeds were germinated on filter paper soaked with sterile water, and the numbers of germinated seeds were counted at 3 days, 4 days, and 5 days after sowing. Data represent the means ± SD of three biological repetitions (n=40), *p < 0.05.

**Figure 5:**
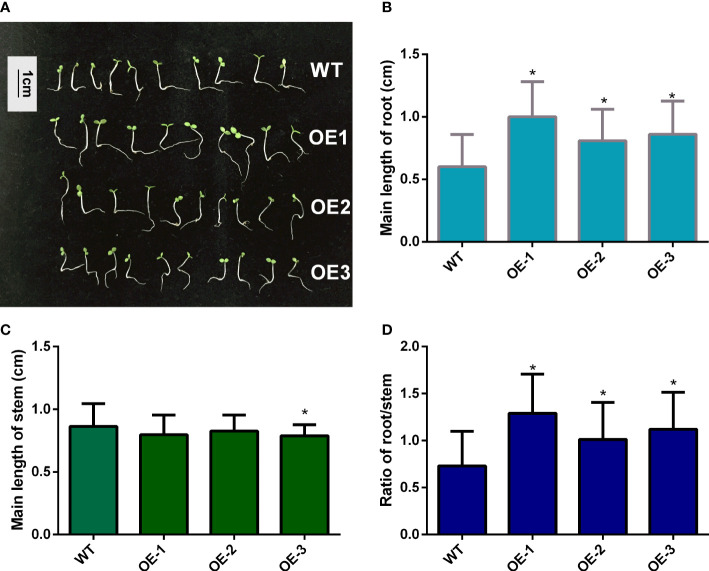
Root and stem length statistics of transgenic tobacco and WT seedlings. **(A)** The phenotype of WT and *MaNCED1* overexpression plants; **(B)** Root and **(C)** stem length of WT and *MaNCED1* overexpression seedlings; **(D)** Ratio of root/stem of WT and *MaNCED1* overexpression seedlings. Seeds germinated on filter paper soaked with sterile water, and the root and stem length were counted 10 days after sowing. Data represent the means ± SD of three biological repetitions (n=10), *p < 0.05.

To determine whether *MaNCED1* is involved in regulating seed germination, the germination rates of wild-type and transgenic lines were counted. The germination rates of transgenic tobacco were significantly lower than that of wild-type (**Figure 4**). Furthermore, we calculated the root and stem lengths of the tobacco seedlings. Compared with the wild type, the root length of transgenic tobacco increased significantly (**Figures 5A, B**). However, the stem length of transgenic plants showed no apparent changes compared with WT (**Figure 5C**). It is worth noting that the ratio of root and stem was significantly increased compared with WT (**Figure 5D**).

### 3.5 The overexpression of *MaNCED1* increases plant tolerance to drought and salt stress

**Figure 6:**
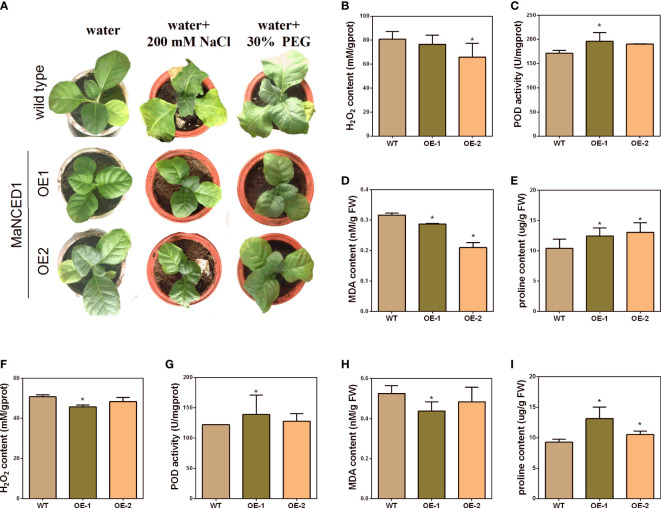
Stress tolerance analyses of *MaNCED1* overexpressed tobacco plants. **(A)** The growth of transgenic tobacco and WT plants under stress conditions. **(B)** The H_2_O_2_ content, **(C)** POD activity, **(D)** MDA content, and **(E)** proline content in *MaNCED1* overexpressed and WT plants under salt stress. **(F)** The H_2_O_2_ content, **(G)** POD activity, **(H)** MDA content, and **(I)** proline content in *MaNCED1* overexpressed and WT plants under drought stress. Data represent the means ± SD of three biological repetitions, *p < 0.05.

**Figure 7:**
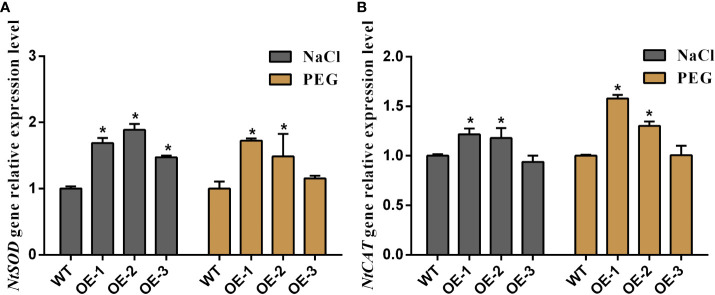
Relative expression levels of **(A)** *NtSOD* and **(B)** *NtCAT* genes in transgenic tobacco and WT plants after NaCl and PEG treatment, respectively. Data represent the means ± SD of three biological repetitions, *p < 0.05.

To visualize the relationship between *MaNCED1* genes and plant stress resistance, five-week-old WT and two transgenic lines (highest and lowerest expressed of *MaNCED1*) were treated with 200 mM NaCl and 30% polyethene glycol (PEG) for 14 d. As shown in **Figure 6A**, the growth of *MaNCED1* overexpression plants is significantly better than that of the wild type (**Figure 6A**). These results suggested that overexpression of *MaNCED1* improved tobacco tolerance to drought and salt stresses. H_2_O_2_, proline, superoxide dismutase (SOD), POD, catalase (CAT), ascorbate peroxidase (APX), and MDA are the key enzymes involved in the scavenging of reactive oxygen species and improving plant resistance under stress conditions (Conde et al., 2011; Hai et al., 2022). The accumulation of H_2_O_2_ and MDA was reduced in *MaNCED1* overexpression plants compared with WT. At the same time, the POD activity and proline content was increased in the transgenic plants after drought and salt treatment (**Figures 6B-G**). Additionally, the expression levels of the superoxide dismutase encoding gene (*NtSOD*) and catalase encoding gene (*NtCAT*) in *MaNCED1* overexpressing tobacco were significantly increased after NaCl or PEG treatment (**Figure 7**).

### 3.6 Overexpression of *MaNCED1* alters the expression of genes involved in seed germination and root growth in tobacco

**Figure 8:**
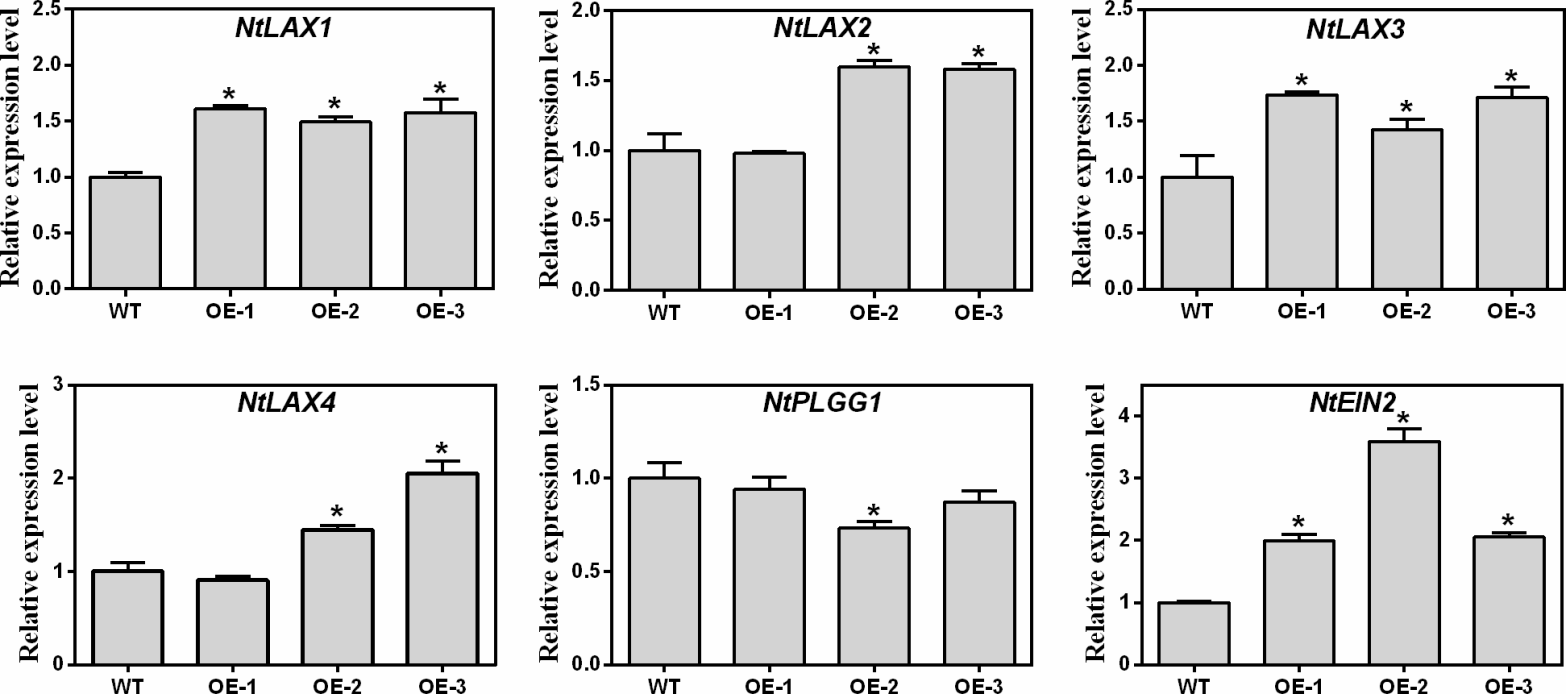
Relative expression levels of genes related to seed germination and root growth in transgenic and WT tobacco. Data represent the means ± SD of three biological repetitions, *p < 0.05.

As shown in **Figure 8**, four auxin transporter-like protein genes (*NtLAX1*, *NtLAX2*, *NtLAX3*, and *NtLAX4*), one plastidal glycolate/glycerate translocator gene (*NtPLGG1*), and one ethylene insensitive 2 (*NtEIN2*) gene which involved in the regulation of seed germination and root growth were selected for analysis. Compared with WT, the expression levels of *NtAUX1*, *NtAUX2*, *NtAUX3*, *NtAUX4*, and *NtEIN2* genes in transgenic tobaccos were increased, while the expression of *NtPLGG1* was slightly decreased (**Figure 8**). Thus, *MaNCED1* might regulate the growth and seed germination by affecting the expression of auxin and ethylene signalling related genes in mulberry.

## 4 Discussion

Salinity and drought are the major abiotic stress influencing the productivity and quality of crops worldwide (Perez-Alfocea et al., 1993; Araújo et al., 2011; Venkatappa et al., 2021; Gonzalez-Villagra et al., 2022). So, it is urgent to characterize salt- and drought-tolerant mechanisms that can be used to develop salt and drought-tolerant crops. As an important plant hormone, ABA regulates plant growth and development, seed germination, and stress resistance (Zhu, 2002; Son et al., 2016). In *Arabidopsis*, the primary root growth was suppressed by ABA treatment (Kim et al., 2016). However, foliage-derived ABA was found to promote root growth and inhibit the development of lateral roots (McAdam et al., 2016). The birch cultured on a medium supplement with ABA increased root length and lateral root number without adversely affecting shoot growth or adventitious root formation (Vaičiukynė et al., 2019). In this study, mulberry seedlings treated with ABA and mannitol significantly increased the ratio of root length to stem length. At the same time, ABA synthesis inhibitors (fluridone and sodium tungstate) and NaCl treatment reduced the root/shoot ratio. However, root and stem growth was inhibited in all these treatments. Longer root length is vital for a plant’s survival from drought stress, allowing the plant to absorb more water. In previous studies, exogenous ABA treatment caused stomatal closure and improved plant tolerance to drought (Li et al., 2020). Overexpression of Gm*CAMTA12* promoted root growth under drought conditions and enhanced drought tolerance in *Arabidopsis* and Soybeans (Noman et al., 2019). This result suggests that ABA regulates mulberry root growth and enhances drought resistance by increasing the root/shoot ratio.

NCED is the key rate-limiting enzyme in the ABA biosynthetic pathway (Huang et al., 2018). Silencing of *NCED* inhibited ABA biosynthesis, reducing ABA accumulation in plants (Gan et al., 2020). In this study, drought and salinity treatment strongly induced the expression of *MaNCED1* in the root. Overexpression of *MaNCED1* in tobacco significantly increased ABA content and improved drought and salt stress tolerance. Similar results have been found in rice and *Arabidopsis* (Iuchi et al., 2001; Estrada-Melo et al., 2015; Hwang et al., 2018). Reactive oxygen species (ROS), indispensable for plant growth and development, are also active in plant resistance to biotic or abiotic stress within limiting and normal concentrations (Choudhury et al., 2017). On the contrary, excessive ROS concentrations can lead to plant cell damage or cell death once the concentrations exceed the scavenging capacity of the plant’s antioxidant system. Various enzymes are involved in scavenging reactive oxygen species and improving plant resistance under stress conditions, such as H_2_O_2_, proline, SOD, POD, CAT, APX, and MDA. They are usually used as indicative parameters for evaluating the oxidative damage of plants under drought and salt stresses (Conde et al., 2011). In this study, overexpressed *MaNCED1* reduced the accumulation of H_2_O_2_ and MDA under drought and salt stress compared with WT. At the same time, the POD activity and proline content was increased in the transgenic plants, suggesting the more comprehensive protection of the transgenic lines from oxidative stress and cell damage. Furthermore, the expression levels of *NtSOD* and *NtCAT* were significantly increased compared with WT, indicating that overexpressing of *MaNCED1* enhanced the tolerance of tobacco to abiotic stresses. Similar results were found in *Arabidopsis*, Malus, and rice (Iuchi et al., 2001; Huang et al., 2018; Hwang et al., 2018).

Dormancy is a crucial process allowing plants to adapt to changing conditions and enables plants to survive under adverse environmental conditions and sustain the species (Wang et al., 2011; Shu et al., 2013). ABA is an important inhibitor during seed germination (Shu et al., 2013). Overexpression of *NCED* promotes ABA accumulation and delays seed germination (Wang et al., 2011), which is consistent with the results of this study. Previous reports indicate that ABA controls root elongation by regulating auxin biosynthesis, distribution and transport (Chen et al., 2016; Fei et al., 2023). High concentrations of exogenous auxin inhibit seed germination in *A. thaliana*, while low concentrations promote seed germination (He et al., 2012). Auxin is required for ABA-mediated inhibition of seed germination, and its deficient mutants show increased resistance to ABA (Thole et al., 2014). Additionally, auxin negatively regulates seed germination and positively regulates seed dormancy (Liu et al., 2013). In this study, *MaNCED1* overexpression leads to increased expression of auxin transporter-like protein genes (*NtLAX1-4*) in tobacco. These results suggested that auxin may coordinate with ABA to inhibit the germination of mulberry seedlings. In addition, evidence indicates that ABA affects root growth and germination and stress response by activating ethylene biosynthesis (Luo et al., 2014). Overexpressing *MaNCED1* in tobacco increases the expression level of *NtEIN2*, implying the crosstalk between ABA and ethylene in regulating plant growth and stress tolerance. PLGG1 encodes a chloroplast protein involved in ABA-inhibited seed germination and drought tolerance (Dong et al., 2018). However, only a slight reduction of *NtPLGG1* was found in transgenic plants. All these results indicate that NCED perhaps affects plant growth and stress response by regulating ethylene and auxin signals.

In conclusion, our results suggest that ABA play an important role in mulberry seedling’s growth. Exogenous ABA treatment inhibited seedling growth but significantly increased the root/stem ratio. Overexpressing *MaNCED1* in tobacco promoted root elongation, inhibited seedings germination and improved salt and drought stress tolerance. ABA might interact with ethylene and auxin to regulate the seed’s germination and abiotic stress tolerance. The present study will provide insights into the functions of NCED from mulberry and other plants in root development and abiotic stress tolerances.

## Data availability statement

The datasets presented in this study can be found in online repositories. The names of the repository/repositories and accession number(s) can be found in the article/**Supplementary material**.

## Author contributions

PZ, CW, and AZ conceived and designed the experiments, PZ, RL, WF, and ZX performed the experiments, PZ, RL, JL, CW, and AZ writing and editing the article. All authors contributed to the article and approved the submitted version.
