# Supplementary material for: A mulberry 9-cis-epoxycarotenoid dioxygenase gene MaNCED1 is involved in plant growth regulation and confers salt and drought tolerance in transgenic tobacco
Source: Front Plant Sci. 2023 Jul 28;14:1228902. doi: 10.3389/fpls.2023.1228902 (PMC10416802; doi:10.3389/fpls.2023.1228902)
Supplement: Supplementary file 1 [file DataSheet_1.docx]

Supplementary Material

A mulberry 9-*cis*-epoxycarotenoid dioxygenase *MaNCED1* is involved in plant growth regulation and confers salt and drought tolerance in transgenic tobacco

**Panpan Zhu, Ruolan Li, Wei Fan, Zhongqiang Xia, Jun Li, Chuanhong Wang*, Aichun Zhao***

**Correspondence:** Corresponding Author: Aichun Zhao: [zhaoaichun@hotmail.com](mailto:zhaoaichun@hotmail.com); [zhaoaichun@swu.edu.cn](mailto:zhaoaichun@swu.edu.cn); Chuanhong Wang: 578748645@qq.com

# Supplementary Figures and Tables

## Supplementary Figures


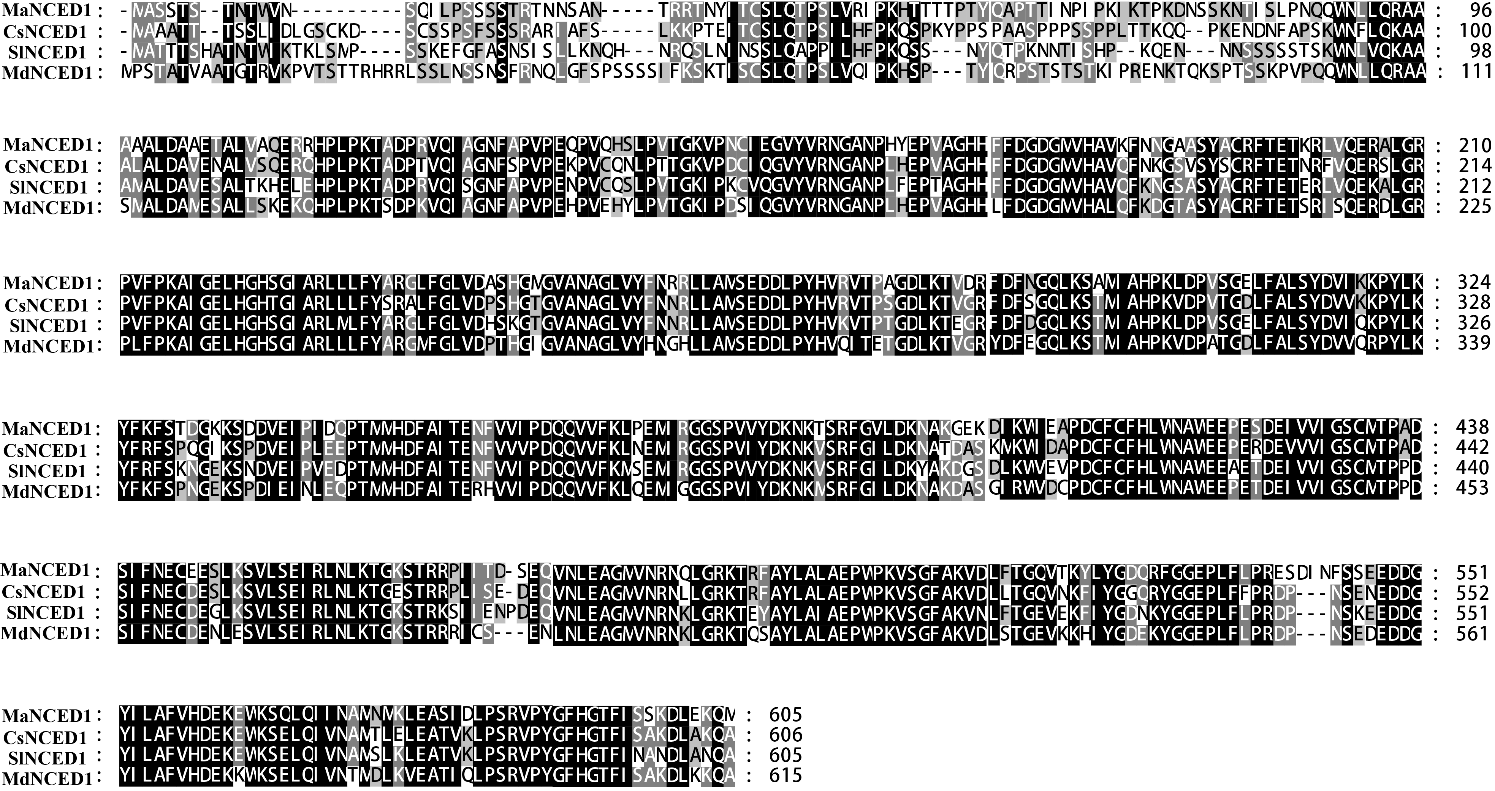


**Supplementary Figure 1.** Multiple sequence alignment of the deduced amino acid sequences of *NCED* genes. Ma: *Morus atropurpurea*; Cs: *Citrus sinensis*; Sl: *Solanum lycopersicum*; Md: *Malus domestica*.


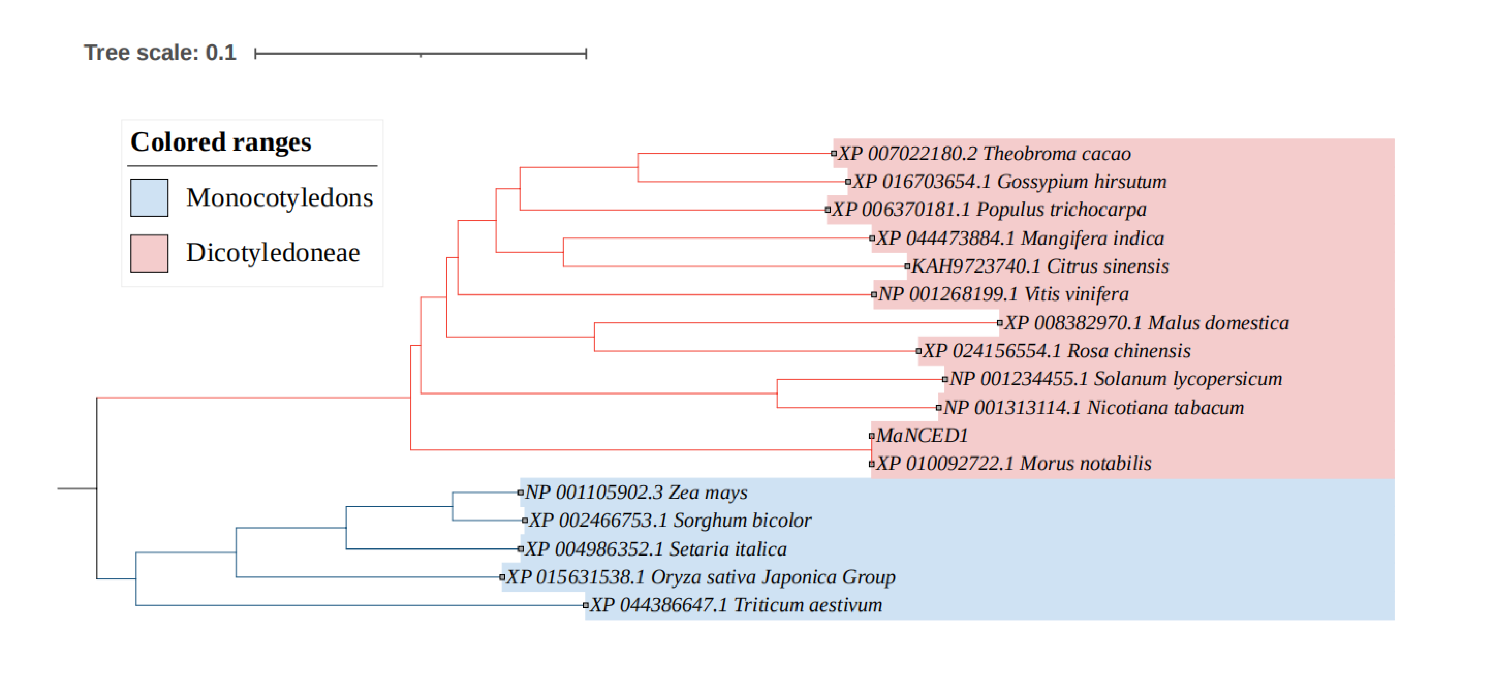


**Supplementary Figure 2.** Phylogenetic analysis of MaNCED1 and other plant NCEDs. The protein sequences were aligned using the CLUSTALW algorithm. A rooted phylogenetic tree was constructed on the basis of the sequence alignment by using MEGA 4.0 software.

**1.2 Supplementary Tables**

**Supplementary Table 1.** Primers were used in this study

| Primer | Primer sequence (5’-3’) | Usage |
| --- | --- | --- |
| *MaNCED1*-F | ATGGCTTCATCAACTTCTAC | Cloning of *MaNCED1* |
| *MaNCED1*-R | TCACATCTGCTTCTCCAAAT |  |
| *OEMaNCED1*-F | CGGGGTACCATGGCTTCATCAACTTCTAC | Overexpression vector construction |
| *OEMaNCED1-*R | CTAGACTAGTTCACATCTGCTTCTCCAAAT |  |
| *NtActin*-F | TCACAGAAGCTCCTCCTAATCCA | Reference gene primer of tobacco |
| *NtActin*-R | GAGGGAAAGAACAGCCTGAATG |  |
| *MaActin* -F | GCATGAAGATCAAGGTGGTG | Reference gene primer of mulberry |
| *MaActin* -R | CATCTGCTGGAAGGTGCTAA |  |
| *MaNCED1*-QF | ATGGACTTGGGCTTCTCATC | qRT-PCR |
| *MaNCED1*-QR | CTGGCTGATTGTAGCGTTGT |  |
| *NtLAX1*-QF | GCGGCGGAGAAACCTCCATT |  |
| *NtLAX1*-QR | GCCCAACCTCCGAACCCAAA |  |
| *NtLAX2-QF* | TGTGGTGGCATGGGTCTTCA |  |
| *NtLAX2-QR* | AGCCTTATGTGGAGGGCATTGA |  |
| *NtLAX3-QF* | TGGGTTCGGAGGTTGGGCTA |  |
| *NtLAX3-QR* | CCGAATGTGGCGGTAGGCT |  |
| *NtLAX4-QF* | GTTGCCGGTGGTGATTCCGA |  |
| *NtLAX4-QR* | CCGCATTCTCTCTGGCAGCA |  |
| *NtPLGG1-QR* | GTGCTCATGGGCTCGGAACA |  |
| *NtPLGG1-QR* | ACTGCGGGAACAGAGCACAC |  |
| *NtEIN2-QR* | TGGGCAACTGGAGTCACCAT |  |
| *NtEIN2-QR* | GGTGGCGCTCGACTACTCAC |  |
